# The impact of stress on sleep quality: a mediation analysis based on longitudinal data

**DOI:** 10.3389/fpsyg.2024.1431234

**Published:** 2024-10-21

**Authors:** Jun Zhang, Shungui Xiang, Xiaowen Li, Yin Tang, Qun Hu

**Affiliations:** ^1^School of Business Administration, Tourism College of Zhejiang, Hangzhou, China; ^2^College of Education, Sehan University, Yeongam County, Jeollanam-do, Republic of Korea; ^3^Library, Tourism College of Zhejiang, Hangzhou, China; ^4^School of Travel Services and Management, Tourism College of Zhejiang, Hangzhou, China

**Keywords:** stress, sleep quality, rumination, social anxiety, Mobile phone addiction, emotion-focused coping strategies

## Abstract

**Objective:**

This study evaluates the potential mechanisms through which stress affects sleep quality and examines the mediating roles of rumination, social anxiety, emotion-focused coping strategies, and smartphone dependence in the relationship between stress and sleep quality.

**Methods:**

From October 2023 to April 2024, we conducted three surveys with 426 university students and utilized structural equation modeling to explore the mechanisms by which stress impacts sleep quality.

**Results:**

Stress not only significantly predicts sleep quality but also significantly affects sleep quality through rumination, emotion-focused coping strategies, and smartphone dependence as independent mediators. Additionally, stress influences sleep quality through both dual-mediation and triple-mediation paths.

**Conclusion:**

Stress has a significant direct and indirect impact on sleep quality. This study reveals the complex mechanisms through which stress affects sleep quality. Improving individuals’ sleep quality requires not only considering the influence of real-life stressors but also examining the intersecting effects between stress and factors such as ruminative thinking, social anxiety, smartphone dependency, and emotion-focused coping strategies. The intense social competition in Chinese society exacerbates the decline in sleep quality, highlighting the need for the government to implement more policies aimed at maintaining the physical and mental health of the population to alleviate the increasingly severe sleep issues and mental health crisis.

## 1 Introduction

Sleep quality refers to an individual’s self-satisfaction with various aspects of their sleep experience. The assessment of sleep quality mainly includes four indicators: sleep efficiency, sleep latency, sleep duration, and early awakening (Wang X. Y. et al., 2023). Factors affecting sleep quality include physiological factors (such as age, circadian rhythms, body mass index, and rapid eye movement sleep), psychological factors (such as stress, anxiety, and depression), and environmental factors (such as room temperature, use of televisions/electronic devices), as well as family/social commitments. Individuals with poor sleep quality are more likely to experience fatigue, irritability, daytime functional impairment, slower reactions, and increased caffeine/alcohol consumption (Nelson et al., 2022).

In 2023, the Institute of Sociology of the Chinese Academy of Social Sciences released the report “China Sleep Research Report 2023.” The sleep quality indicator used in this survey was the sleep index, with a score of 60 as the baseline for acceptable sleep quality; higher scores represent better sleep quality. Data showed that the overall sleep index for residents in China was 67.77, slightly above the acceptable threshold of 60. Longitudinal data in the report indicated a general decline in sleep duration and quality with age. Gender comparisons showed little difference in average sleep duration between males and females, though males had slightly higher sleep index scores than females. Higher-educated individuals (with postgraduate education or above) tended to have shorter sleep durations and significantly lower sleep scores compared to those with lower education levels (He et al., 2023). In 2024, the Institute of Sociology of the Chinese Academy of Social Sciences released the results of the 2023 National Resident Sleep Quality Survey, showing that the overall sleep index for residents in China was 62.61, a decrease of 5.16 points from the previous year. This indicates a remarkably rapid decline in the sleep index score in just 1 year (Wang et al., 2024). In fact, in China, short sleep duration and poor sleep quality have become prevalent and increasingly concerning health issues, possibly related to the high stress resulting from rapid economic and social development (Yuan and Xing, 2016).

A large-scale survey of Chinese adults found that over 40% of respondents reported getting less than 7 h of sleep per night, and nearly 60% indicated that they frequently or occasionally experienced poor sleep quality issues, such as difficulty falling asleep, waking up during the night, or waking up early (Li et al., 2018). Additionally, the study discovered that individuals with poor sleep quality often face a range of health problems, including anxiety, depression, decreased cognitive function, and an increased risk of cardiovascular diseases (Xu et al., 2016). These issues not only impact individuals’ physical and mental health but can also negatively affect work efficiency, social functioning, and overall quality of life. Why is sleep quality so poor among Chinese people? Jones and Brighton (2018) suggest that high levels of work-related stress are a significant factor contributing to reduced sleep duration and poor sleep quality.

Stress is a psychological and physiological response that individuals experience when dealing with challenges, demands, or uncertainties from their internal or external environments. This response can include emotional symptoms such as anxiety and tension, as well as physiological changes like increased heart rate and muscle tension (Song et al., 2022). The factors influencing stress are multifaceted, including individual characteristics, environmental factors, and coping strategies. Regarding individual characteristics, factors such as personality traits, coping abilities, and social support can affect how one perceives and manages stress. Environmental factors, such as stressors related to work, education, and family, can also impact individuals (Segerstrom and Smith, 2019; Slavich, 2020). Long-term exposure to stress can lead to psychological health issues such as anxiety and depression, characterized by emotional instability, negative self-evaluation, and difficulties in coping (Choi S. W. et al., 2019). This, in turn, can trigger physiological stress responses, including the release of hormones like adrenaline and cortisol, potentially affecting physiological functions such as the cardiovascular, immune, and digestive systems. This may increase the risk of cardiovascular diseases, diabetes, and other chronic conditions (Alonso et al., 2021). Individuals who face prolonged stress might engage in unhealthy behaviors such as increased consumption of high-calorie foods, excessive alcohol intake, and smoking. These behaviors may be related to stress-induced emotional dysregulation, psychological health issues, and changes in coping strategies (Choi K. W. et al., 2019). Stress can also impact sleep quality, leading to insomnia or insufficient sleep, which in turn affects physical health and cognitive function (Altena et al., 2020). Additionally, stress can impair cognitive functions such as attention, memory, and decision-making abilities, potentially reducing work and study efficiency (Shields et al., 2016).

The Stress-Sleep Relationship Theory Model posits that stressors can impact sleep quality, with variations depending on the duration and intensity of the stress. Additionally, acute versus chronic and positive versus negative stress can exhibit different characteristics and outcomes. Decreased sleep quality can also become a stressor, creating a feedback loop where sleep quality affects and is affected by stress. Beyond direct impacts, the role of mediating factors such as stress coping and stress responses is also crucial (Yan et al., 2010). Stress can lead to increased release of physiological hormones like adrenaline and cortisol, affecting sleep regulation centers and cognitive functions, which in turn results in reduced sleep quality (Altena et al., 2020). The research tool used in this study to measure sleep quality is the Epworth Sleepiness Scale. A higher score on this scale indicates poorer nighttime sleep quality and greater daytime sleepiness. Therefore, this study proposes the following hypothesis:

*Hypothesis 1:* Stress can significantly predict sleep quality.

Some researchers argue that the mechanisms through which stress impacts sleep quality are complex and require a comprehensive assessment of factors that may affect sleep. Generally, under long-term stress conditions, individuals may develop various psychological disorders, which further trigger negative emotional experiences (such as social anxiety) (Yan Y. et al., 2013), negative behavioral responses (such as emotion-focused coping strategies) (Zhang Y. et al., 2023), psychological internal conflicts (such as rumination) (Lin H. et al., 2023), and unhealthy addictive behaviors (like Mobile phone addiction) (Wan and Ding, 2024a). Stress can lead individuals to experience rumination, a cognitive pattern where individuals repeatedly recall, ponder, and struggle to break free from issues or dilemmas (Lin J. et al., 2023). This cognitive pattern exacerbates negative emotions (such as social anxiety), causing discomfort in social interactions (Yan H. et al., 2013), increasing emotional stress, and disrupting normal sleep patterns (Tampke E. C. et al., 2019). Additionally, stress often prompts individuals to adopt emotion-focused coping strategies, such as avoidance, denial, or self-blame (Zhang Y. F. et al., 2023). Using smartphones is one way to temporarily escape from real-life stress, as frequent smartphone use seeks psychological comfort (Wan and Ding, 2024b). However, these coping strategies typically fail to effectively resolve problems and may instead worsen the individual’s psychological burden, leading to a decline in sleep quality (Cedeno D. et al., 2023; Guan L. et al., 2020). Therefore, in this study, we use social anxiety, emotion-focused coping strategies, Mobile phone addiction, and rumination as mediating variables to explore the relationship between stress and sleep quality.

### 1.1 Mediating effects of rumination, social anxiety, emotion-focused coping strategies, and Mobile phone addiction

#### 1.1.1 Mediating effect of rumination

Rumination is a cognitive process where individuals repeatedly revisit, ponder, and analyze problems or difficulties, finding it challenging to escape this cycle (Li et al., 2022). This type of thinking often manifests as repetitive reflection and deep contemplation of negative events or emotions, leading individuals to experience states of brooding, worry, or depression (Lin H. et al., 2023). The biopsychosocial model emphasizes that an individual’s health and well-being are influenced by a combination of biological factors (including genetics, physiology, biochemistry, and any conditions or diseases affecting physical health), psychological factors (including cognitive processes, emotions, personality traits, coping mechanisms, and mental health conditions such as depression or anxiety, as well as beliefs, attitudes, and stress responses that might affect health outcomes), and social factors (including relationships, family dynamics, socioeconomic status, cultural background, community support, access to healthcare, and environmental influences). The complex interplay of these biological, psychological, and social factors often significantly impacts overall health (Ghaemi, 2018). This theory has been further validated in practice, showing significant relationships among stress, rumination, and sleep quality. Specifically, stress is significantly positively correlated with rumination, while stress and rumination are both significantly negatively correlated with sleep quality (Fang et al., 2023; Chen et al., 2022; Zhu et al., 2020). Stress from work and life events can alter physiological hormone levels, leading to psychological states such as anxiety and depression, which negatively impact individuals’ physiological, psychological, and social well-being, disrupting normal sleep and reducing sleep quality (Wan and Ding, 2024a; Guan Y. Q. et al., 2020).

Currently, there is relatively limited research on the relationships between stress, sleep quality, and rumination in humans, but we can explore these relationships through related studies in animals. In the field of animal husbandry, researchers have found that stress may indirectly affect rumination behavior in ruminant animals by influencing physiological and behavioral processes, and that the emotions and sleep behaviors of ruminants are often affected by their rumination behavior (Rosenbaum et al., 2018). Therefore, we might infer that stress could affect sleep quality by influencing rumination behavior in ruminants. However, whether these findings in animals apply to humans still requires further validation. Based on this, we propose.

*Hypothesis 2:* Rumination significantly mediates the relationship between stress and sleep quality.

#### 1.1.2 Mediating effect of social anxiety

Social anxiety is a common psychological disorder in clinical practice, characterized by excessive worry, fear, or discomfort in social or public situations involving interactions with others or self-presentation (Liu et al., 2024). This anxiety may be related to fears of negative evaluation by others, concerns about one’s actions or performance leading to disapproval or ridicule, which often results in individuals avoiding social situations or feeling extremely uncomfortable in them. Social anxiety is frequently accompanied by physical symptoms such as increased heart rate, trembling hands, and sweating, as well as psychological symptoms like feelings of inferiority and self-criticism, causing significant distress and suffering (Richards et al., 2015). The biopsychosocial model posits that social anxiety results from the complex interplay of biological, psychological, and social factors. Biological factors, such as genetic predispositions and neurochemical imbalances, may increase sensitivity to social situations. Psychological factors, including negative self-perceptions and lack of social skills, may heighten the risk of social anxiety. Social factors, such as environmental stress and social experiences, also influence an individual’s level of social anxiety (Ghaemi, 2018). Research has found a positive correlation between stress and social anxiety, as well as a significant negative correlation between stress and sleep quality. Specifically, greater interpersonal stress is associated with more severe social anxiety and a notable decline in sleep quality (Chen et al., 2022; Siegel et al., 2018).

Other studies have also identified a significant relationship between social anxiety and sleep quality, suggesting that anxiety-induced cognitive disruptions may impair sleep onset (Yan X. et al., 2013; Tampke D. et al., 2019). Based on this, the present study proposes the following hypothesis:

*Hypothesis 3:* Social anxiety mediates the relationship between stress and sleep quality.

#### 1.1.3 Mediating effect of emotion-focused coping strategies

Emotion-focused coping strategies refer to behaviors or psychological states where individuals use negative, avoidant, or helpless approaches that are not conducive to problem-solving or emotional regulation when faced with stress, challenges, or difficulties (Everett et al., 2004). These strategies include avoiding problems, denying difficulties, self-blame, and dwelling on negative emotions. Such approaches typically lead to the persistence or worsening of problems and a decline in the individual’s mental health (Zhang Y. F. et al., 2023). The Vulnerability-Stress-Adaptation (VSA) Model posits that individuals under constant stress, especially those with poor adaptability, often resort to emotion-focused coping strategies. These are short-term maladaptive strategies such as avoidance, denial, and self-blame (Cedeno A. et al., 2023). A study investigating the stress and emotion-focused coping strategies among 120 tuberculosis patients found that their sources of stress included the disease itself, hospital environment, and societal factors. In terms of coping strategies, these patients primarily used avoidance and resignation, and there was a significant positive correlation between stress and emotion-focused coping strategies (Yang K. Z. et al., 2021).

The stress coping model suggests that individuals exhibit different emotional and behavioral responses when facing stress, which can lead to various types of mental disorders, such as sleep disorders (Morin et al., 2003). Zhang et al. (2018) conducted a study with university nursing students to explore the relationships between sleep quality, coping strategies, and depressive symptoms. The results revealed that individuals using active coping strategies, especially those engaging in active problem-solving and emotional regulation, might mitigate the impact of declining sleep quality on depressive symptoms to some extent. In contrast, individuals employing emotion-focused coping strategies often experienced a significant decline in sleep quality (Zhang et al., 2018). Based on this, the present study proposes the following hypothesis:

*Hypothesis 4:* Emotion-focused coping strategies mediate the relationship between stress and sleep quality.

#### 1.1.4 The mediating role of Mobile phone addiction

Mobile phone dependency refers to excessive reliance on smartphones or mobile phones, characterized by overuse, excessive attention, and emotional or behavioral dependence on the device (An and Jiang, 2020). This dependency can lead to ineffective control over phone use in daily life, affecting normal functioning in work, study, social interactions, and even having negative impacts on mental health (Panova and Carbonell, 2018). The Interaction of Person-Affect-Cognition-Execution (I-PACE) model suggests that individuals may engage in addictive behaviors (such as drug addiction, phone dependency, gaming, etc.) as a way to cope with stress from negative experiences. Behavioral addiction can lead to manifestations similar to substance addiction, including cravings, uncontrollable use, and functional impairment due to continued use, and in some cases, the behavior becomes automatic and habitual (Brand et al., 2019; Roberts et al., 2017; Schneider et al., 2017; Starcke et al., 2018).

According to the “Statistical Report on China’s Internet Development,” published by the China Internet Network Information Center, by June 2021, the number of mobile internet users in China had reached 1.16 billion, accounting for 82.0% of the total population. This indicates that the majority of Chinese people are accustomed to accessing the internet via their mobile phones. With the widespread adoption of smartphones, an increasing number of people have developed the habit of using their phones before bedtime. The blue light emitted by phones can affect the secretion of melatonin, disrupt natural sleep rhythms, and the psychological stimulation from phone use can also make it difficult to fall asleep (Wang, 2014). Empirical research supports these findings, showing a significant positive correlation between stress and phone use. When individuals face higher levels of stress, especially when feeling overwhelmed, they tend to unconsciously choose to escape from real-life stress, often through means such as alcohol addiction, phone addiction, or drug addiction (Zhang, 2015). Prolonged phone use is associated with a noticeable decline in sleep quality, with significant negative correlations between phone use and sleep quality, as well as between stress and sleep quality (Li et al., 2016a,b; Zhang B. et al., 2021; Zhang D. et al., 2021). Based on this, the study proposes the following hypothesis:

*Hypothesis 5:* Mobile phone dependency mediates the relationship between stress and sleep quality.

### 1.2 Relationships among rumination, social anxiety, emotion-focused coping strategies, and Mobile phone addiction

#### 1.2.1 The relationship between rumination and social anxiety

Rumination and social anxiety are closely related. Cognitive models of social anxiety suggest that social anxiety arises from individuals’ negative cognitive patterns and thought processes about social situations, such as excessive concern about others’ evaluations, underestimating their own abilities, and over-worrying about negative outcomes. Cognitive theories emphasize the importance of altering these negative cognitive patterns to reduce social anxiety. They view biases in interpreting ambiguous social cues as a key process in maintaining social anxiety. For example, a socially anxious person might interpret someone yawning during a conversation as a sign of boredom rather than fatigue, leading to increased state anxiety (Beard and Amir, 2010).

Rumination is a negative cognitive process where individuals repeatedly revisit and reflect on negative experiences, worries, and self-criticism from social situations, which exacerbates anxiety and fear. This thinking pattern is often closely associated with social anxiety and may be a significant feature of social anxiety disorder. Rumination is considered a crucial influencing factor for symptoms of depression and social anxiety (Kross et al., 2014). It can not only intensify depressive emotions but also impair an individual’s problem-solving abilities, making it difficult to effectively address challenges. This thinking pattern can trap individuals in a cycle of negative emotions, preventing them from finding solutions to their problems (Spinhoven et al., 2018). Research has found a positive correlation between rumination and social anxiety, meaning that greater rumination is associated with higher levels of anxiety in social situations. Reducing rumination can effectively alleviate symptoms of social anxiety disorder and improve quality of life (Nordahl et al., 2016).

#### 1.2.2 The relationship between rumination, Mobile phone addiction, and emotion-focused coping strategies

Compensatory Internet Use Theory posits that if individuals feel discomfort in their real-life environment, they may use mobile phones to escape from real-life difficulties and meet unmet needs, potentially leading to excessive mobile phone dependence or internet addiction (Kardefelt-Winther, 2014). Zhang B. et al. (2021) conducted a survey among Chinese college students and found that female students scored significantly higher than males on mobile phone dependence overall, as well as on the sub-factors of inefficacy and escapism. Mobile phone dependence scores were significantly positively correlated with sleep quality, sleep onset latency, sleep disturbances, and daytime dysfunction scores, and also showed a significant positive correlation with rumination. Higher levels of mobile phone dependence make it easier for individuals to detach from their real-life environment, reducing actual behavioral engagement. Consequently, they are more likely to experience frustration in real life, which can increase psychological stress such as depression and anxiety. Repeated obsessive thinking and reflection on personal experiences during the night can lead to more active thinking and difficulty falling asleep (Zhang B. et al., 2021).

Rumination is a negative cognitive process characterized by repeatedly thinking about, recalling, and emphasizing negative experiences, emotions, and issues. Emotion-focused coping strategies, on the other hand, are a range of unhealthy, ineffective, or avoidant methods used to deal with stress, challenges, or negative emotions. It is easy to understand that individuals engaged in rumination may generate negative emotions (Kross et al., 2014), which can lead them to adopt more emotion-focused coping behaviors, such as avoiding difficulties, denying problems, or indulging in negative emotions. Conversely, emotion-focused coping strategies may also exacerbate the frequency and intensity of rumination, creating a vicious cycle. This cycle traps individuals in a deeper emotional and psychological distress, gradually worsening their overall well-being.

#### 1.2.3 The relationship between emotion-focused coping strategies, Mobile phone addiction, and social anxiety

Approach–avoidance theory suggests that although emotion-focused coping strategies may temporarily alleviate negative emotions, they do not improve an individual’s ability to effectively solve problems or face challenges. Therefore, in the long term, emotion-focused coping strategies may actually exacerbate the severity of the issues (Corr and Krupi, 2017). Mobile phone dependence is a form of escape behavior resulting from stress, where individuals use their phones to avoid or distract themselves from difficult situations in real life (Lin, 2018). A study investigated the relationship between Mobile phone addiction and adolescent mental health, with a focus on the association between Mobile phone addiction and emotion-focused coping strategies. The results showed a significant positive correlation between Mobile phone addiction and emotion-focused coping strategies. Adolescents with higher levels of Mobile phone addiction were more likely to use emotion-focused coping strategies (such as avoidance and denial) to cope with challenges and stress. They might be more prone to escaping real-life problems by immersing themselves in phone use, thereby avoiding negative emotions and challenges, which creates a negative cycle (Choi K. W. et al., 2019). This association not only has the potential to negatively impact adolescents’ mental health but may also lead to reduced engagement in important activities such as social interactions, learning, and work, increasing the likelihood that they will adopt emotion-focused coping strategies (Elhai et al., 2017; Hawi and Samaha, 2016).

Cognitive models of social anxiety suggest that individuals tend to use emotion-focused coping strategies to manage stress and discomfort in social situations. However, these coping methods often exacerbate anxiety and make it difficult for individuals to escape the anxious state. For instance, avoiding social situations might lead to a decline in social skills and confidence, which in turn increases social anxiety levels. Research has found a significant positive correlation between emotion-focused coping strategies and social anxiety (Liao et al., 2014; Beard and Amir, 2010). A study assessing 2,695 Chinese college students found that autistic traits and emotion-focused coping strategies were positively correlated with social anxiety, with emotion-focused coping strategies mediating the relationship between autistic traits and social anxiety (Yang T. Y. et al., 2021). Based on this, the following hypotheses are proposed:

*Hypothesis 6:* Rumination and social anxiety mediate the relationship between stress and sleep quality.

*Hypothesis 7:* Rumination and mobile phone dependence mediate the relationship between stress and sleep quality.

*Hypothesis 8:* Emotion-focused coping strategies and mobile phone dependence mediate the relationship between stress and sleep quality.

*Hypothesis 9:* Rumination – Emotion-focused coping strategies – Mobile phone dependence mediate the relationship between stress and sleep quality.

*Hypothesis 10:* Rumination – Emotion-focused coping strategies – Social anxiety mediate the relationship between stress and sleep quality.

*Hypothesis 11:* Rumination – Social anxiety – Mobile phone dependence mediate the relationship between stress and sleep quality.

## 2 Methods

### 2.1 Participants

We used a cluster sampling method to select three universities from a total of 20 higher education institutions in Anhui and Zhejiang provinces in China. The selected universities were Tourism College of Zhejiang, Bozhou University, and Zhejiang Police Vocational College. All enrolled students from these institutions who had no classes on the day of the survey participated in the study, with no exclusion criteria applied. We strictly adhered to the relevant provisions of the Helsinki Declaration, and after obtaining approval from the Ethics Committee of Tourism College of Zhejiang, we proceeded with the questionnaire survey. This study required three rounds of longitudinal data collection, with a three-month interval between each survey. The first survey was conducted on October 17, 2023, with 438 questionnaires distributed and collected; the second survey was conducted on January 19, 2024, with 426 questionnaires distributed and collected; and the third survey was conducted on April 22, 2024, with 440 questionnaires distributed and collected.

Due to the fact that a small number of students were absent during the three rounds of the questionnaire surveys, and since this study is longitudinal, we only retained data from participants who completed all three surveys. After excluding invalid responses, we ultimately obtained 426 valid questionnaires. Of these, 77 participants (18.1% of the total) were boys, and 349 participants (81.9% of the total) were girls. Additionally, 326 participants (76.5% of the total) were from rural areas, while 100 participants (23.5% of the total) were from urban areas. We extracted stress data as the independent variable from the first survey, rumination, social anxiety, mobile phone dependence, and emotion-focused coping strategies data as mediating variables from the second survey, and sleep quality data as the dependent variable from the third survey. Prior to the survey, we emphasized the purpose of the study and the voluntary nature of participation, and we obtained written informed consent from the students. No compensation was provided to participants. The questionnaire surveys were conducted in classrooms, with all participants independently completing the demographic variables and the questionnaires on stress, sleep quality, rumination, social anxiety, emotion-focused coping strategies, and mobile phone dependence. After completion, the questionnaires were collected and verified by the examiner.

### 2.2 Research instruments

#### 2.2.1 College student stress scale

We used the stress scale for Chinese college students developed by Li and Mei (2002) to measure stress levels. This scale consists of 30 items covering three dimensions: academic stress (e.g., “Despite efforts, my grades in some subjects are still poor”), personal stress (e.g., “Desiring true love but not receiving it”), and negative life events (e.g., “Failing more than two courses”). The scale employs a 4-point Likert scoring method, where participants choose from 0 (no stress) to 3 (severe stress). Higher scores indicate greater perceived stress at the current time (Li and Mei, 2002). The scale in this study demonstrated excellent internal reliability (Cronbach’s *α* = 0.97) and structural validity: Comparative Fit Index (CFI) = 0.94, Tucker-Lewis Index (TLI) = 0.92, Root Mean Square Error of Approximation (RMSEA) = 0.07, and Standardized Root Mean Square Residual (SRMR) = 0.04.

#### 2.2.2 Epworth sleepiness scale

The Chinese version of the Epworth Sleepiness Scale, developed by Johns, was used. This version was tested among Chinese populations using the scale revised by Chinese scholars Mei et al., which demonstrated good reliability and validity. The scale is primarily used to assess an individual’s sleep quality (Mei et al., 2024). The scale contains only 8 items and employs a 4-point Likert scoring method, where participants are asked to choose from 0 (never) to 3 (high chance). Higher total scores indicate a stronger tendency for daytime sleepiness. The scoring interpretation is as follows: 0–9 points: Normal sleep quality; 10–15 points: Mild decrease in sleep quality, leading to low daytime sleepiness; 16–24 points: Moderate decrease in sleep quality, leading to moderate daytime sleepiness; 25 points and above: Extremely poor sleep quality, leading to severe daytime sleepiness (Scharf, 2022; Johns, 1991). The scale in this study demonstrated good internal reliability (Cronbach’s *α* = 0.86) and structural validity: Comparative Fit Index (CFI) = 0.93, Tucker-Lewis Index (TLI) = 0.90, Root Mean Square Error of Approximation (RMSEA) = 0.08, and Standardized Root Mean Square Residual (SRMR) = 0.05.

#### 2.2.3 Mobile phone addiction scale

The initial mobile phone dependency scale was developed by Leung based on the diagnostic criteria for addiction outlined in the Diagnostic and Statistical Manual of Mental Disorders, Fourth Edition (DSM-IV) to measure the extent of Mobile phone addiction. We used the Chinese version revised by Shi et al. (2016) to ensure the measurement tool is suitable for Chinese students. This scale includes 17 items covering four dimensions: Withdrawal (e.g., “You start to feel anxious about missing calls if you do not check your phone for an extended period”). Loss of Control (e.g., “Your friends and family complain that you always use your phone”). Inefficiency (e.g., “You delay other activities because you are busy using your phone, causing trouble”). Escape (e.g., “You have used your phone to relieve feelings of sadness when you felt down”). The scale uses a 5-point Likert scoring method, where participants are asked to choose from 1 (not at all) to 5 (always). Higher scores indicate higher levels of addiction (Wang, 2022; Leung, 2008). The scale in this study demonstrated good internal reliability (Cronbach’s *α* = 0.95) and structural validity: Comparative Fit Index (CFI) = 0.93, Tucker-Lewis Index (TLI) = 0.92, Root Mean Square Error of Approximation (RMSEA) = 0.07, and Standardized Root Mean Square Residual (SRMR) = 0.05.

#### 2.2.4 Rumination scale

We used the Rumination Scale developed by Nolen-Hoeksema, which was revised for this study to ensure that the measurement tool is suitable for the Chinese population. This scale measures the level of rumination and consists of three dimensions with a total of 12 items. These dimensions are: Symptom Rumination Dimension: Includes 3 items (e.g., “I often think about why I feel so unhappy”). Reflective Rumination Dimension: Includes 5 items (e.g., “I often think about what I did that led to this”). Compulsive Reflection Dimension: Includes 4 items (e.g., “I often think alone about why this happened”). The scale uses a 4-point Likert scoring method, where participants are asked to choose from 1 (never think about depressing things) to 4 (always think about depressing things) (Wang H. et al., 2023; Nolen-Hoeksema, 2000). The scale in this study demonstrated good internal reliability (Cronbach’s *α* = 0.96) and structural validity: Comparative Fit Index (CFI) = 0.96, Tucker-Lewis Index (TLI) = 0.95, Root Mean Square Error of Approximation (RMSEA) = 0.07, and Standardized Root Mean Square Residual (SRMR) = 0.03.

#### 2.2.5 Social anxiety scale

We used the Chinese version of the Social Anxiety Scale initially developed by Fenigstein et al., with revisions completed by You to better suit the measurement of social anxiety levels in the Chinese population (You, 2024). This scale consists of a single factor with a total of 6 items. Item 4, “I find it easy to talk to strangers,” is a reverse-scored item, while the remaining 5 items are scored positively. The scale uses a 4-point Likert scoring method. For example, for item 2, “I find it difficult to work when someone is watching me,” participants are asked to choose from 0 (not at all like me) to 3 (very much like me). A higher score indicates a higher level of social anxiety (Kai and Jin, 2022; Fenigstein et al., 1975). In this study, the scale demonstrated good internal reliability (Cronbach’s *α* = 0.84) and structural validity: Comparative Fit Index (CFI) = 0.94, Tucker-Lewis Index (TLI) = 0.91, Root Mean Square Error of Approximation (RMSEA) = 0.08, and Standardized Root Mean Square Residual (SRMR) = 0.03.

#### 2.2.6 Emotion-focused coping strategies questionnaire

The original questionnaire on coping strategies was developed by Folkman and Lazarus, and Fang et al. revised it for Chinese adolescents to create the Chinese version of the Coping Strategies Questionnaire (Fang et al., 2018). This scale consists of 20 items across two factors: Positive Coping Strategies and Emotion-Focused Coping Strategies. The Positive Coping dimension (e.g., “seeking advice from relatives, friends, or classmates”) includes 12 items, while the Emotion-Focused Coping Strategies dimension (e.g., “using smoking, drinking, medication, or eating to relieve distress”) includes 8 items. Participants are asked to choose from a scale ranging from 0 (not at all) to 3 (very often) (Ye, 2023; Folkman et al., 1988). In this study, we will use the Emotion-Focused Coping Strategies dimension as the measurement tool for emotion-focused coping strategies. The scale demonstrates good internal reliability (Cronbach’s *α* = 0.85) and structural validity: Comparative Fit Index (CFI) = 0.90, Tucker-Lewis Index (TLI) = 0.92, Root Mean Square Error of Approximation (RMSEA) = 0.08, and Standardized Root Mean Square Residual (SRMR) = 0.05.

### 2.3 Data analysis strategy

We utilized SPSS 25.0 software to calculate the mean, standard deviation, and Pearson correlation coefficients for stress, sleep quality, rumination, social anxiety, Emotion-Focused Coping Strategies, and Mobile phone addiction. Confirmatory factor analysis (CFA) was performed using Mplus 7.0 software to assess the internal structural validity of the survey instruments used in this study. Maximum likelihood estimation was employed to address missing data in the questionnaires. Following the confirmation of adequate fit for the structural validity indicators of the stress, sleep quality, rumination, social anxiety, Emotion-Focused Coping Strategies, and Mobile phone addiction questionnaires, we constructed a structural equation model to evaluate its validity. Model fit indices were assessed based on the Comparative Fit Index (CFI), Tucker-Lewis Index (TLI), Root Mean Square Error of Approximation (RMSEA), and Standardized Root Mean Square Residual (SRMR). The criteria proposed by Brown (2015) were applied to determine model fit adequacy, with a model considered to have a reasonable fit if RMSEA <0.1, SRMR <0.1, TLI > 0.9, and CFI > 0.9 (Ji et al., 2023).

**Figure 1:**
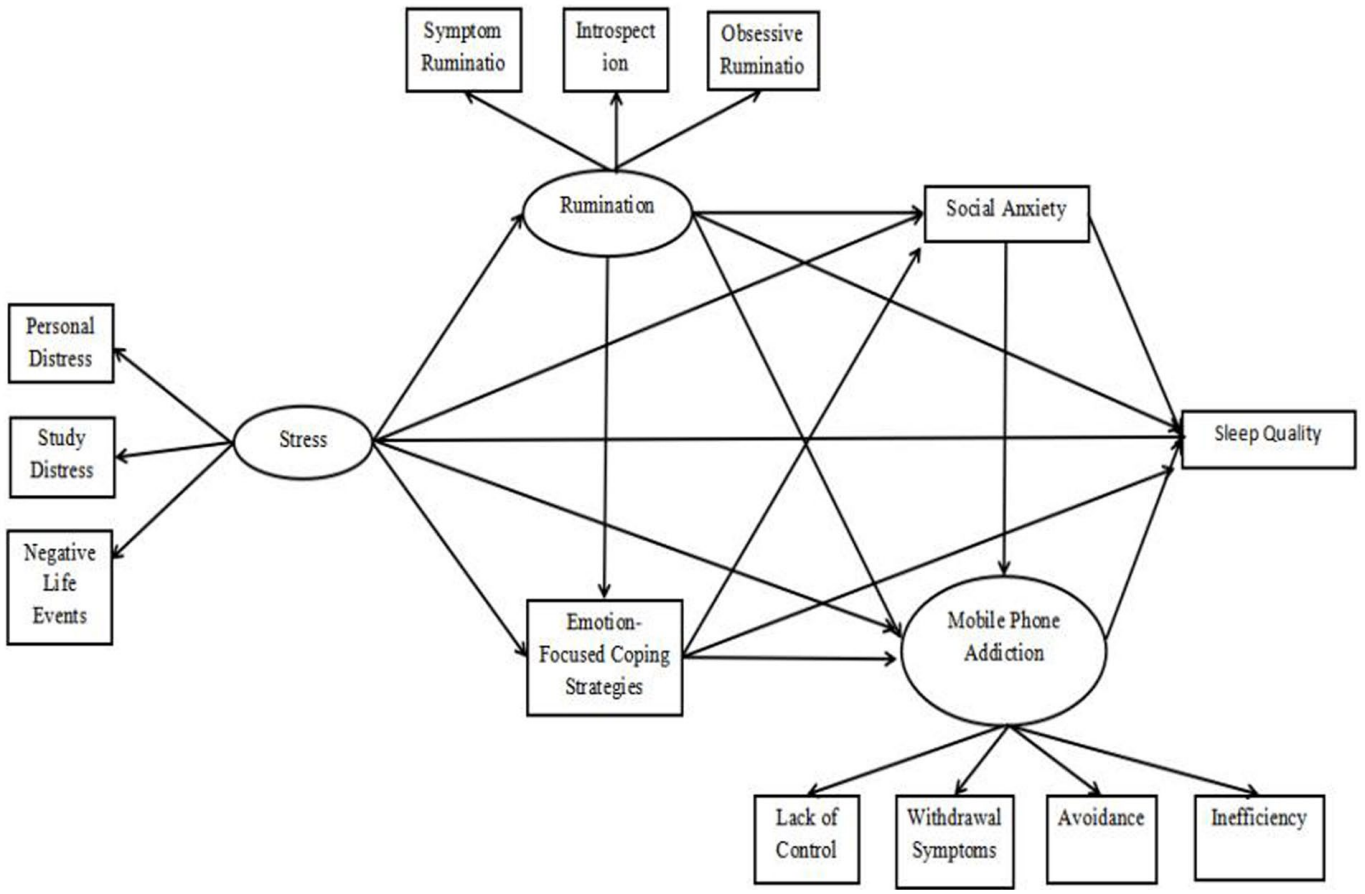
Indirect effects model of stress on sleep quality.

The research was conducted in two phases. First, a direct relationship model was established with stress as the independent variable and sleep quality as the dependent variable. Second, an indirect relationship model was developed where stress serves as the independent variable, and sleep quality is the dependent variable, with rumination, social anxiety, Emotion-Focused Coping Strategies, and Mobile phone addiction acting as mediating variables, as illustrated in Figure 1. To assess the significance of the indirect effects, Bootstrap 95% confidence intervals for bias-corrected bootstrapped tests were employed. Indirect effects were deemed significant if the Bootstrap 95% confidence intervals did not include zero.

## 3 Results

### 3.1 Control and verification of common method bias

To address the issue of common method bias, we implemented various measures, including the use of anonymous questionnaires and reverse-scored items. Exploratory factor analysis was conducted using Harman’s single-factor method. The results indicated that 11 factors with eigenvalues greater than 1 accounted for 70.45% of the total variance. Additionally, the variance explained by the first principal factor was 39.50%, which is below the 40% threshold (He et al., 2022), suggesting that substantial common method bias is not present.

### 3.2 Descriptive statistics and correlation analysis

We utilized the independent sample *t*-test method to investigate the variances in demographic variables related to household registration and gender concerning Stress, Sleep quality, rumination, social anxiety, Emotion-Focused Coping Strategies, and Mobile phone addiction. Statistically significant differences were identified between male and female participants in the scores of social anxiety and Mobile phone addiction (*p* < 0.05), whereas no significant distinctions were noted in Stress, Sleep quality, rumination, and Emotion-Focused Coping Strategies scores (*p* > 0.05). Significant differences were observed between participants from urban and rural households regarding Stress, social anxiety, and rumination scores (*p* < 0.05), whereas no statistically significant variances were evident in Sleep quality and Emotion-Focused Coping Strategies scores (*p* > 0.05) (refer to Table 1). Additionally, significant positive correlations were identified among the six variables—Stress, Sleep quality, rumination, social anxiety, Emotion-Focused Coping Strategies, and Mobile phone addiction—suggesting interrelatedness among them. The means, standard deviations, and correlation coefficients for each variable are detailed in Table 2.

**Table 1:** Differences in stress, sleep quality, rumination, social anxiety, emotion-focused coping strategies, and Mobile phone addiction among different genders and registered residences.

Dependent variable	Independent variable	*F*	Significance	*t*	Sig (two-tailed)
Sleep quality	Gender	4.99	0.02	−1.54	0.12
Social anxiety		0.19	0.66	−2.79	0.00
Emotion-focused coping strategies		8.50	0.00	−1.93	0.05
Rumination		9.91	0.00	−0.37	0.70
Stress		12.35	0.00	−0.86	0.38
Mobile phone addiction		6.69	0.01	−3.09	0.00
Sleep quality	Household registration	0.00	0.93	1.10	0.26
Social anxiety		3.58	0.05	2.26	0.02
Emotion-focused coping strategies		0.05	0.81	1.58	0.11
Rumination		1.22	0.26	1.97	0.04
Stress		1.34	0.24	3.09	0.00
Mobile phone addiction		1.55	0.21	1.57	0.11

**Table 2:** Means, standard deviations, and correlation coefficients of variables.

	1	2	3	4	5	6	7	8
1. Gender	1							
2. Household registration	0.04	1						
3. Sleep quality	0.07	−0.05	1					
4. Social anxiety	0.13**	−0.10*	0.37**	1				
5. Emotion-focused coping strategies	0.09	−0.07	0.49**	0.50**	1			
6. Rumination	0.01	−0.09*	0.50**	0.52**	0.53**	1		
7. Stress	0.04	−0.14**	0.52**	0.50**	0.54**	0.69**	1	
8. Mobile phone addiction	0.14**	−0.07	0.46**	0.48**	0.54**	0.55**	0.56**	1
Quantity			426	426	426	426	426	426
Minimum value			12.00	0.00	17.00	6.00	0.00	0.00
Maximum value			48.00	90.00	85.00	24.00	24.00	24.0
Mean			21.90	23.67	44.91	13.84	8.37	7.63
Standard deviation			6.51	15.30	13.40	3.66	4.31	4.10

*N* = 426. * *p* < 0.05, ** *p* < 0.01, *** *p* < 0.001.

### 3.3 Direct effect analysis

We constructed a direct relationship model with Stress as the independent variable and Sleep quality as the dependent variable to investigate whether Stress can directly predict Sleep quality. Path analysis demonstrated that Stress significantly and positively predicted Sleep quality (*β* = 0.53, *p* < 0.001), thus confirming Hypothesis 1.

### 3.4 Mediation analysis

**Figure 2:**
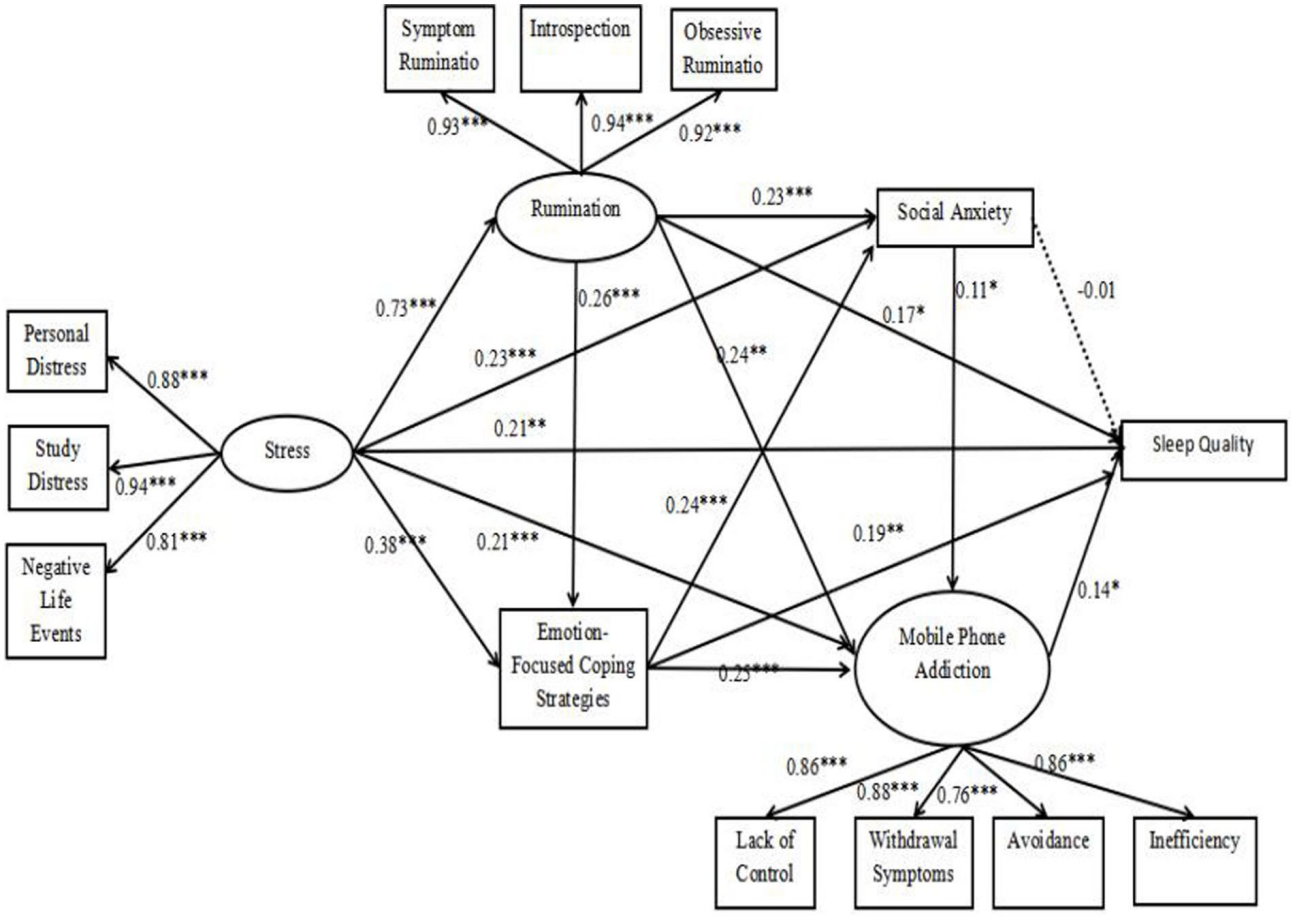
Mediation model of the effect of stress on sleep quality.

Due to the large number of items in the stress scale, rumination scale, and mobile phone dependency scale used in this study, we utilized the item packaging method for modeling (Wu and Wen, 2011). Based on the direct relationship model mentioned above, we inserted four mediators (i.e., rumination, social anxiety, emotion-focused coping strategies, and mobile phone dependency) between stress and sleep quality to establish an indirect relationship model (see Figure 2). The results show that the model fit indices are satisfactory: RMSEA = 0.05, SRMR = 0.04, TLI = 0.96, CFI = 0.97.

To evaluate the significance levels of the indirect relationships shown in Figure 2, we used Bootstrap resampling with 1,000 iterations to test the chain mediation effects and calculate 95% confidence intervals. The results indicated that among the 10 mediation paths we constructed in the indirect relationship model, 7 paths had Bootstrap 95% confidence intervals that did not include zero, suggesting that these 7 mediation effects were significant. Specifically, Hypotheses 2, 4, 5, 7, 8, and 9 were supported. In contrast, the remaining 3 mediation paths had Bootstrap 95% confidence intervals that included zero, indicating that these 3 mediation effects were not significant (see Table 3). Thus, Hypotheses 3, 6, and 10 were not supported.

**Table 3:** Bootstrap analysis for significance testing of mediation effects.

Mediation pathway	Effect size	95% Confidence interval	
		Lower bound	Upper bound
Stress-rumination-sleep quality	0.07	0.01	0.14
Stress-social anxiety-sleep quality	0.00	−0.02	0.01
Stress-emotion-focused coping strategies-sleep quality	0.04	0.01	0.08
Stress-mobile phone addiction-sleep quality	0.01	0.00	0.05
Stress-rumination-social anxiety-sleep quality	0.00	−0.01	0.01
Stress-rumination-mobile phone addiction-sleep quality	0.01	0.00	0.03
Stress-emotion-focused coping strategies-mobile phone addiction-sleep quality	0.00	0.00	0.01
Stress-rumination-emotion-focused coping strategies-mobile phone addiction-sleep quality	0.00	0.00	0.01
Stress-rumination-emotion-focused coping strategies-social anxiety-sleep quality	0.00	0.00	0.00
Stress-rumination-social anxiety-mobile phone addiction-sleep quality	0.00	0.00	0.00

## 4 Discussion

This study found that stress can significantly predict sleep quality. According to social cognitive theory, the interactions among individuals, their environment, and the interplay between individuals and their environment are crucial for behavioral outcomes. The uniqueness of each situation—considering the environment, participants, and their interactions—often leads to different learning, behavioral expressions, and outcomes (Beauchamp et al., 2019). Stress can lead to negative cognitive activities such as worry, anxiety, and rumination, which can interfere with the ability to fall asleep and maintain sleep, thereby affecting sleep quality (Kyle et al., 2015; Pa et al., 2015). In real life, the high-pressure lifestyle in China (including work stress, academic pressure, and social stress) and the competitive social environment often result in symptoms of anxiety and depression. The prevalence of stress disorders, obsessive-compulsive disorder, social phobia, anxiety, and depression is increasing annually among the Chinese population, with generally low sleep quality and prevalent sleep issues such as difficulty falling asleep, shallow sleep, and nighttime awakenings. These issues significantly impact individuals’ quality of life and health (Li et al., 2018), posing a major challenge to mental health (Wang et al., 2014; Huang et al., 2019). In this study, we also found that ruminative thinking, emotion-focused coping strategies, and smartphone dependency significantly impact sleep quality, while social anxiety does not have a direct significant effect on sleep quality. This result suggests that for individuals to achieve sustainable development, it is important to address the pressures and sleep quality issues they face. For example, individuals should learn techniques such as deep breathing, meditation, and progressive muscle relaxation to help them relax before sleep and reduce the negative impacts of stress on their physical and mental well-being.

We found that stress mediates sleep quality through two dual mediation paths. First, stress mediates sleep quality through the mediation path of “emotion-focused coping strategies – mobile phone dependency.” The approach–avoidance theory suggests that when individuals face stress, they may adopt negative coping strategies such as avoidance, denial, or immersing themselves in negative emotions (Corr and Krupi, 2017). Prolonged use of mobile phones, especially before bedtime, can lead to exposure to blue light, which suppresses melatonin production and interferes with falling asleep and sleep quality. Additionally, mobile phone use may also trigger excitement and arousal, making it difficult for individuals to relax and fall asleep. When sleep quality deteriorates, individuals’ coping abilities may weaken, making them more prone to falling into a cycle of emotion-focused coping strategies and mobile phone dependency (Levenson et al., 2016). Secondly, stress mediates sleep quality through the mediation path of “rumination – mobile phone dependency.” Response style theory suggests that when facing negative emotions or stress, individuals may become trapped in a cycle of rumination. Rumination involves the repetitive, deep thinking, and analysis of negative experiences, emotions, or problems. Individuals often attempt to understand, resolve, or escape stress and negative emotions through rumination. However, rumination typically exacerbates emotional discomfort and psychological distress, leading individuals into a cycle of negative emotions, which increases their perception of stress and psychological burden (Black and Possel, 2015). Individuals may use mobile phones excessively as a means of escaping reality or regulating negative emotions. Mobile phone use provides a way to escape reality and regulate emotions, temporarily relieving individuals from stress and negative emotions (Elhai et al., 2017). The longer the phone use, the greater the phone dependency, and the worse the sleep quality (Demirci et al., 2015). In today’s Chinese society, people spend a significant portion of their lives using electronic devices (such as smartphones and computers). Although this usage often arises from work-related needs, it can have a substantial negative impact on people’s lives and is detrimental to their overall development.

We found that stress mediates sleep quality through three intermediary paths. First, stress mediates sleep quality via “rumination-emotion-focused coping strategies-smartphone dependence.” When individuals experience high levels of stress, their ability to manage negative emotions may weaken, making them more prone to entering a state of rumination (Smith et al., 2008). This excessive focus on negative emotions leads to a greater tendency to use emotion regulation strategies when facing emotional distress. Such selective attention can hinder effective problem-solving, resulting in coping mechanisms that focus more on emotional management rather than problem resolution (Nolen-Hoeksema, 2000), potentially causing long-term emotional distress (Nolen-Hoeksema, 2001). Over-reliance on short-term emotional relief methods, such as smartphone dependence, social media, or engaging in other highly stimulating activities (like gaming or browsing news), can trigger psychological and physiological arousal, leading to a sustained state of excitement and difficulty relaxing before sleep (Rosen et al., 2013). Secondly, stress mediates sleep quality through the “rumination-social anxiety-smartphone dependence” pathway. In high-stress environments, an individual’s cognitive resources and emotional regulation abilities may be impaired, leading to increased frequency and intensity of rumination (Robinson and Alloy, 2003). Research indicates that this thought pattern causes individuals to constantly recall and amplify their negative experiences in past social situations, increasing anxiety about future social events (Moulds and Wong, 2009). Additionally, rumination may make individuals overly sensitive to their performance in social interactions, further exacerbating symptoms of social anxiety (Rood et al., 2011). Smartphones offer a relatively safe means of communication, allowing them to engage socially in a controlled environment and reducing opportunities to confront social anxiety directly (Elhai et al., 2017). This may lead to excessive time spent on smartphones, increasing smartphone dependence. Browsing social media and receiving notifications at night can also disrupt sleep, reducing the amount of deep sleep individuals get (Liu et al., 2017) and causing delays in falling asleep and nighttime awakenings (Demirci et al., 2015). Third, the level of social anxiety is positively correlated with the severity of rumination. Individuals with social anxiety tend to exhibit higher levels of rumination, often repeatedly recalling and analyzing negative experiences or concerns from social interactions (McEvoy and Brans, 2013). The more severe the rumination (Aldao et al., 2014), the more likely these individuals are to adopt negative coping strategies when faced with problems (Aldao et al., 2010), which makes them more susceptible to excessive smartphone use (Elhai et al., 2017). To my knowledge, there is currently limited research in academia on how stress mediates sleep quality through multiple intermediary paths. Based on the findings of this study, we need to recognize that the mechanisms by which stress affects sleep quality are complex. Stress may exert its influence through independent mediating variables, as well as through mediation paths involving two variables (such as “rumination and smartphone dependence” and “emotion-focused coping strategies and smartphone dependence”) or three variables (such as “rumination-emotion-focused coping strategies-smartphone dependence,” “rumination-social anxiety-smartphone dependence,” and “rumination-emotion-focused coping strategies-social anxiety-smartphone dependence”). Given the numerous factors influencing sleep quality, individuals can effectively improve their sleep by maintaining a regular sleep schedule, increasing moderate physical activity, establishing relaxing pre-sleep habits (such as deep breathing and meditation), and managing stress and anxiety.

## 5 Limitations and implications

We must acknowledge some limitations in this study. Firstly, with only 426 participants, the sample size is relatively small, which may affect the generalizability of the results to other populations. Secondly, although the self-report scales used in this study demonstrated internal reliability and validity, there remains uncertainty about whether the data accurately reflects the participants’ psychological states. Therefore, future research should incorporate multiple methods to ensure the quality of the research.

Despite these limitations, this study not only supports the approach–avoidance theory and social cognitive theory but also empirically validates these theories. Additionally, by collecting longitudinal data, the results of this study are more robust. Furthermore, this research provides a new framework for understanding the relationship between stress and sleep quality, offers new insights for improving sleep quality in psychological health practices, and contributes new knowledge to the theoretical and empirical research on the relationship between stress and sleep quality.

## 6 Conclusion

Stress not only significantly impacts sleep quality directly but also has a substantial effect on sleep quality through dual and triple mediation paths formed by rumination, social anxiety, emotion-focused coping strategies, and smartphone dependence. Given the severe challenges faced by the sleep quality of individuals in China, it is essential for labor protection departments to promote the formulation and implementation of policies that support healthy sleep, such as providing appropriate rest breaks in the workplace. Additionally, stress management and relaxation techniques, such as deep breathing, meditation, or yoga, can be promoted to help alleviate stress and anxiety, thereby improving sleep quality.

## Data Availability

The datasets presented in this study can be found in online repositories. The names of the repository/repositories and accession number(s) can be found at: zhangjunahnu@163.com. If you request, I can provide to you.
